# SIRT2 Affects Cell Proliferation and Apoptosis by Suppressing the Level of Autophagy in Renal Podocytes

**DOI:** 10.1155/2022/4586198

**Published:** 2022-04-22

**Authors:** Shuang Liu, Xiangfu Gao, Zhenliang Fan, Qiao Wang

**Affiliations:** ^1^Geriatrics, The Third Affiliated Hospital of Zhejiang Chinese Medical University, Hangzhou, China; ^2^Nephrology, The Third Affiliated Hospital of Zhejiang Chinese Medical University, Hangzhou, China; ^3^Nephrology, The First Affiliated Hospital of Zhejiang Chinese Medical University, China; ^4^Department of Rheumatology, Zhejiang Chinese Medical University, Hangzhou, China

## Abstract

**Purpose:**

Despite the discovery of many important molecules in diabetic nephropathy, there has been very limited progress in the management of diabetic kidney diseases and the design of new drugs. To fill this gap, the present study explored the expression of SIRT2 in high-glucose murine kidney foot cells and its impact on cell biological functions.

**Methods:**

Expression levels of SIRT2 in the MPC-5 of murine kidney foot cells after high and normal glucose treatment or in cells targeted with siRNA were detected using qRT-PCR. Cellular proliferation and programmed cell death were analyzed via the CCK8 assay and flow cell technique, separately. Levels of autophagy markers were measured by western blotting, and chloroquine treatment was applied to the cells to observe the effect of SIRT2 on cell proliferation and apoptosis after treatment.

**Results:**

The expression level of SIRT2 was remarkably upregulated in the high-GLU group in contrast to the low-GLU group. The cell proliferation and autophagy levels were significantly reduced, and apoptosis was remarkably reinforced in the high-GLU group in contrast to the normal GLU group. However, knocking down the expression level of SIRT2 caused an increase in cell proliferation and cell autophagy levels and significantly weakened apoptosis. Chloroquine influenced cell proliferation and apoptosis in cells targeted with SIRT2 siRNA.

**Conclusion:**

SIRT2 expression was upregulated in hyperglycaemic murine kidney foot cells, and knocking down the expression level of SIRT2 affected the biological function of the cells. We found that SIRT2 may modulate cell proliferation and apoptosis by regulating cell autophagy.

## 1. Introduction

Diabetes is a systemic disease in which the body is unable to produce or respond effectively to the glucose-regulating hormone insulin. The World Health Organization estimates that approximately 422 million people have diabetes, and that the quantity of diabetic patients is on the rise [1]. Diabetes mellitus can be classified into type 1 diabetes, which is induced by idiopathic autoimmunity destruction of pancreas *β*-cells, and T2D, which stems from widespread peripheral INS resistance [2]. Hyperglycaemia disrupts hemodynamic and metabolic homeostasis, and the chronic nature of this microenvironmental imbalance promotes the development of diffuse cellular abnormalities [3].

One of the most serious consequences of diabetes is the development of diabetic vascular disease, which manifests clinically as microvascular and macrovascular complications, with diabetic nephropathy being a key microvascular complication of diabetes [4]. Diabetic nephropathy affects 30–45% of sufferers with T1D or T2D, with the morbidity peaking during the 10–20 years of diabetes [5]. As with many renal diseases, diabetic nephropathy is characterised by the presence of proteinuria, which is caused by apoptosis and loss of podocyte function, followed by a decrease in glomerular filtration rate associated with glomerulosclerosis [6]. The treatment of diabetic nephropathy is primarily concerned with preventing or delaying disease progression. Despite the discovery of many important molecules in hypothesis-driven studies over the last two decades, there has been very limited progress in the management of diabetic kidney diseases and in the design of new drugs.

Sirtuin 2 (SIRT2) is a histone deacetylase, which deacetylates tubulin, AKT, and other proteins [7]. SIRT2 is primarily localised in the cytoplasm, deacetylates histones, and many nonhistone proteins and plays a key role in various physiological processes [8]. Currently, there is increasing evidence that the aberrant expression of SIRT2 may be associated with human diseases, neurological disorders, and cancer [9]. For example, SIRT2 expression is increased in acute myeloid leukaemia mother cells, and downregulating SIRT2 expression promotes apoptosis in HeLa cells [10]. SIRT2 is upregulated in hepatocellular carcinoma tissues compared to adjacent normal tissues, while migration and invasion of human HCC cells are reduced by knocking down SIRT2 [11]. However, the effect of SIRT2 on diabetic nephropathy has been less studied clinically. In this research, the expressing of SIRT2 was observed, and its effect on the biological function of the cells was investigated by subjecting murine kidney foot cells MPC-5 to low- and high-GLU treatments.

## 2. Materials and Methods

### 2.1. Main Materials and Instruments

The following items were used in this study: mouse renal pedal cell MPC-5 (Xiamen Yimu Biotechnology Co., Ltd., Item No. IM-M011), real-time fluorescence quantitative PCR instrument (ABI, Model No. QuantStudio 6), Trizol reagent (Ambion, Item No. 15596-026), HiScript Reverse Transcriptase (VAZYME, Model No. R101-01/02), microspectrophotometer (Hangzhou Aosheng Instruments Co., Ltd., Model No. Nano-100), RIPA lysis solution (Biyuntian, Item No. P0013B), BCA protein concentration determination kit (Biyuntian, Item No. P0013B), RIPA lysis solution (Biyuntian, Item No. P0013B), BCA protein concentration determination kit (Biyuntian, Item No. P0010), PVDF membrane (Millipore, Item No. IPVH00010), rabbit multiple antibody SIRT2 (42KD, Affinity, Item No. DF6076), HRP-labeled sheep anti-rabbit second antisubstance (Wuhan Ph. Ltd., Item No. BA1054), rabbit multiple anti-GAPDH (37KD, Hangzhou Xianzhi Biological Co., Ltd., Item No. AB-P-R 001), ECL substrate solution (Beijing Pulilai Gene Technology Co., Ltd., Item No. P1050), rabbit multiple anti-LC3 (14/16KD, Wuhan Sanying Biotechnology Co. AP), rabbit multiple antibodies P62 (62KD, Wuhan Sanying Biotechnology Co., Ltd., Item No. 18420-1-AP), RPMI 1640 medium (GIBCO, Item No. 11875-093, sugar-free Item No. 11879020), glucose (Sigma, Item No. G7528), Lipofectamine 2000 (Invitrogen, Item No. 11668), and rabbit multiple antibodies LC3 (14/16KD, Wuhan Sanying Biotechnology Co. Invitrogen, Item No. 11668-019), siRNA (Hanheng Bio), CO_2_ incubator (SANYO, Model No. MCO-15AC), low-speed centrifuge (Eppendorf, Model No. 5702R), enzyme marker (Molecular Devices (MD), Model No. Flexstation® 3), CCK8 cell proliferation and cytotoxicity assay kit (MCE, Item No. HY-K0301), flow cytometer (Beckman-Coulter, Model No. cytoFLEX), inverted microscope (OLYMPUS, Model No. IX51), apoptosis detection kit (Nanjing KGI Biological, Item No. KGA108), paraformaldehyde (Sinopharm, item number: 80096618), DAPI (Biyuntian, item number: C1002), and antifluorescence quenching blocker (SouthernBiotech, item number: 0100-01). Primer synthesis was performed using DynaScience (Tables 1 and 2).

## 3. Methods

### 3.1. Cell Culture, Grouping, and Transfection

The routine passaged culture was performed using a high sugar RPMI 1640 medium with 10% FBS solution+1% PNC/kyowamycin solution, and the growth conditions were in a cellular cultivation device under 37°C at 5% carbon dioxide. After the cells were cultivated in the logarithmic growth phase, the cultivation intermediary was substituted by RPMI 1640 intermediary without serum for subsequent transfection. Transfection of cell lines used Lipofectamine^TM^ 2000 transfection kit according to kit instructions and transfected separately according to the following experimental groups: MPC5+NG (blank plasmid group) (5.5 mM D-GLU), MPC5+HG (30 mM D-GLU), MPC5+NG+NC, MPC5+HG+NC, MPC5+NG+siRNA1, MPC5+NG+siRNA2, MPC5+NG+siRNA3, MPC5+NG+SIRT2 siRNA2, MPC5+HG+SIRT2 siRNA2, MPC5+HG+NC+10 *μ*M chloroquine, and MPC5+HG+SIRT2 siRNA2+10 *μ*M chloroquine. The HG group was treated with high glucose for 24 h before transfection, and MPC5+HG+NC+10 *μ*M chloroquine and MPC5+HG+SIRT2 siRNA2+10 *μ*M chloroquine were treated with 10 *μ*M chloroquine for 3 h after 45 h of high-glucose treatment. Cells were collected 48 h after transfection for subsequent experiments. Each experiment was repeated three times.

### 3.2. qRT-PCR Assay

We added 1 ml of Trizol reagent to the cellular suspending liquid strictly according to the instructions. The mixture was mixed well with a gun, moved to a 1.5 ml EP tube without RNase, and allowed to be lysed for 10 min. Total RNA was extracted, and OD260, OD280, and OD260/OD280 results were determined with a microspectrophotometer to calculate the purity and content of RNA. The quality of RNA was speculated according to the OD260/OD280 rate, which was between 1.8 and 2.0. The specimen RNA content was calculated as per the equation below based on the absorbance value: total RNA concentration (*μ*g/*μ*l) = OD260 × 40 × 10^−3^. The mRNA was reverse transcribed using HiScript Reverse Transcriptase to synthesise the cDNA reverse transcriptional reaction system (20 *μ*L): 5 *μ*g RNA, 2 *μ*L Oligo (dT) 18 (10 *μ*M), 4 *μ*L dNTP (2.5 mM), 4 *μ*L 5× HiScript Buffering solution, 1 *μ*L HiScript reversed transcriptive enzyme, and 0.5 *μ*L RNase suppressor and added ddH_2_O up to 20 *μ*L. The reaction status were stated below: 25°C for 5 minutes, 50°C for 15 minutes, 85°C for 5 minutes, 4°C for 10 minutes, and 5 minutes, 4°C for 10 minutes. The qRT-PCR reaction system was as follows: 0.2 *μ*L forward primer (10 *μ*M), 0.2 *μ*L reverse primer (10 *μ*M), 5 *μ*L SYBR Green Master Mix, 0.2 *μ*L 50× ROX referential dye 2, and 0.2 *μ*L H_2_O. The reactive conditions were as follows: 50°C for 2 minutes, 95°C for 10 minutes, 95°C for 0.5 minutes, and 60°C for 0.5 minutes for an overall 40 cycles. The dissolution curves were plotted, and final data were analysed as 2^-△△Ct^.

### 3.3. Western Blot (WB) Assay

The transfected cells were lysed by RIPA lysis solution, and protein content was detected via a BCA kit. The protein content was modified, subjected to separation via 12% SDS-PAGE electrophoretic method, and moved onto 0.45 *μ*m PVDF membrane after ionisation. The film was incubated in TBST with 5% nonfat milk powder (closure solution) on a shaking device under RT for 2 h. SIRT2 1 : 1000 or LC3 1 : 1000 and P62 1 : 1000 were supplemented and cultivated nightlong under 4°C. The PVDF film was cleaned five times with TBST for 5 min/time. The relevant HRP-labeled second antisubstance was desaturated with TBST–1 : 50,000 dilution, and the PVDF film was placed into the second antisubstance cultivation solution and cultivated for 120 min under RT in a shaking device. The PVDF film was cleaned well again with TBST five times (5 min/time). The enhanced liquor in the ECL reagent with the steady peroxide enzyme liquor was mixed at a proportion of 1 : 1, and the working liquor was supplemented to the PVDF film drop by drop. After a few minutes or until the fluorescent band was obvious, the excess substrate solution was aspirated with a filter paper. The X-ray film was developed, scanned, and analysed with BandScan.

### 3.4. CCK8 Detection of Cell Proliferation

The transfected cells were made into a suspension and inoculated into 96-well plates with 100 *μ*L/well of cell suspension, respectively. Three replicate wells were set up in each well, and 10 *μ*L CCK8 was supplemented to every well and cultivated under 37°C for 4 h. The OD result of every group of cells was subsequently measured under an absorbance of 450 nm using an enzyme marker.

### 3.5. Apoptosis by Flow Cytometry

Cells were subjected to digestion with 0.25% tryptic enzyme with no EDTA and collected after the termination of digestion, centrifuged at 1500 rpm for 300 s, and the supernatant was removed and resuspended with PBS. The cells were cleaned for two times in PBS under 1500 rpm for 300 s. We supplemented 500 *μ*l binding buffer to resuspend the cells, then mix with 5 *μ*l AnnexinV-FITC and add 5 *μ*l propidium iodide, mix and react for 5~15 min at room temperature and avoid light, and finally detect apoptosis on the flow cytometer.

### 3.6. Confocal Observation

In the culture plate, the slides with crawling cells were cleaned in PBS three times for 180 s every time. The crawling slides were subjected to fixation in 4% PFA for 15 min, and the slides were cleaned in PBS three times for 180 s every time. The specimens were incubated with DAPI dropwise for 300 s in the dark, and the excess DAPI was washed off using PBST four times for 300 s. The crawling slides were blotted with absorbent paper, and the slides were sealed with blocking solution containing antifluorescent reagent. The images were then collected under a fluorescence microscope.

## 4. Statistics

The measurement data are presented as average ± SD, and the method for comparing data between several groups is one-way analysis of variance. Post hoc two-by-two comparisons were made by LSD-*t*-test, and the data between two groups were tested by independent samples *t*-test. Diversities had significance when *p* < 0.05. The entire statistic analysis and plots were performed via Prism 8 program (GraphPad).

## 5. Results

### 5.1. SIRT Family Expression in High and Normal Glucose Levels

**Figure 1:**
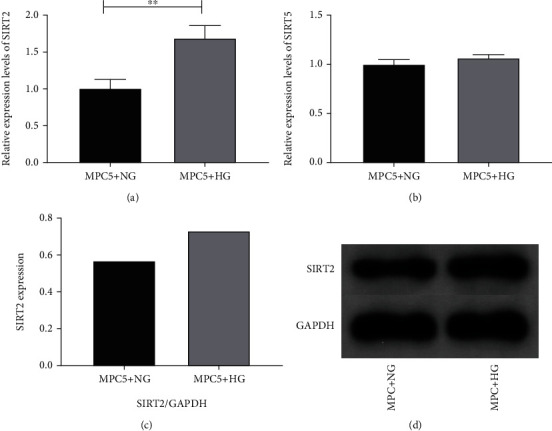
SIRT family expression in excessive and normal GLU levels. (a) Expression of SIRT2 in excessive and normal GLU levels. (b) Expression of SIRT5 in high and normal glucose levels. (c) WB indicates SIRT2 protein levels. (d) SIRT2 protein expression graph. Note: ^∗∗^*p* < 0.01 for the contrast between groups.

We subjected mouse kidney podocytes MPC-5 to high or normal glucose treatment and observed the expression of SIRT2 and SIRT5 at high and normal glucose levels. The outcomes revealed that the expressing level of SIRT2 was significantly upregulated in excessive GLU in contrast to normal GLU status (*p* < 0.05). However, the levels of SIRT5 in excessive and normal GLU conditions were not remarkably diverse (*p* > 0.05). WB also revealed that the expressing of SIRT2 was upregulated in excessive GLU conditions in MPC-5 cells (Figure 1).

### 5.2. Effect of Knockdown of SIRT2 Expression Levels on Cell Proliferation

**Figure 2:**
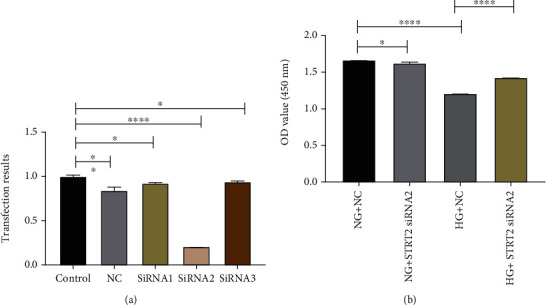
Effect of knocking down the expression level of SIRT2 on cell proliferation. (a) SIRT2 interference with siRNA screening. (b) Effect on cell proliferation after knockdown of SIRT2 expression level. Note: ^∗^*p* < 0.05 for the contrast between groups; ^∗∗^*p* < 0.01 for the contrast between groups; ^∗∗∗∗^*p* < 0.0001 for the contrast between groups.

We used siRNA1, siRNA2, and siRNA3 to interfere with SIRT2 expression, and the results showed that siRNA2 transfection efficiency was the best. Thus, siRNA2 was chosen for subsequent interference experiments in which we observed the effect of knocking down SIRT2 on cell proliferation. The results showed that cell proliferation was significantly reduced at excessive GLU levels in contrast to normal GLU levels. However, by interfering with the SIRT2 expression level, we showed that the cell proliferation was increased at high glucose (*p* < 0.05) (Figure 2).

### 5.3. Effect of Knockdown of SIRT2 Expression Levels on Apoptosis

**Figure 3:**
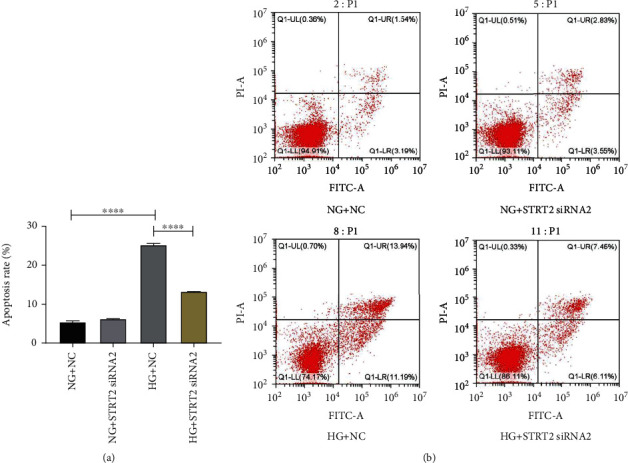
Effect of knocking down the expressing level of SIRT2 on apoptosis. (a) Effect of knockdown of SIRT2 expression level on apoptosis rate. (b) Flow cytogram of apoptotic cells in each group. Note: ^∗∗∗∗^*p* < 0.0001 for comparison between groups.

We also observed the effect of SIRT2 on apoptosis by modulating its expression level. The results showed that apoptosis was remarkably reinforced at high-GLU conditions in contrast to normal GLU conditions. However, by interfering with the expression level of SIRT2, we showed that apoptosis was significantly weakened at high glucose (*p* < 0.05) (Figure 3).

### 5.4. High Levels of SIRT2 Expression Affect the Level of Cellular Autophagy

**Figure 4:**
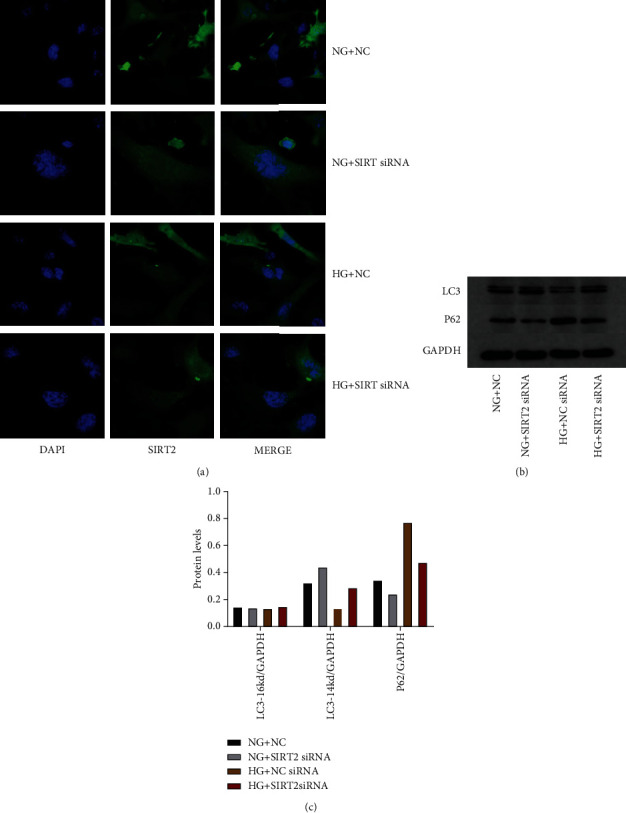
SIRT2 expressing level affects the level of cellular autophagy. (a) Confocal micrographs of each group. (b) Expression of autophagy-associated proteins. (c) Histogram of the expression of autophagy-related proteins.

We assessed the effect of a high SIRT2 expressing level on the level of cellular autophagy. The results showed that there was a remarkable drop in LC3 levels and a significant elevation in P62 levels in high-glucose conditions, both of which could indicate that the autophagy level of cells was significantly reduced at high glucose. However, after SIRT2 expression was knocked down, there was an elevation in LC3 level and a drop in P62 level at high glucose, which could indicate that the autophagy level of cells increased when SIRT2 was knocked down (*p* < 0.05) (Figure 4).

### 5.5. SIRT2 Affects Cell Proliferation by Regulating Cell Autophagy

**Figure 5:**
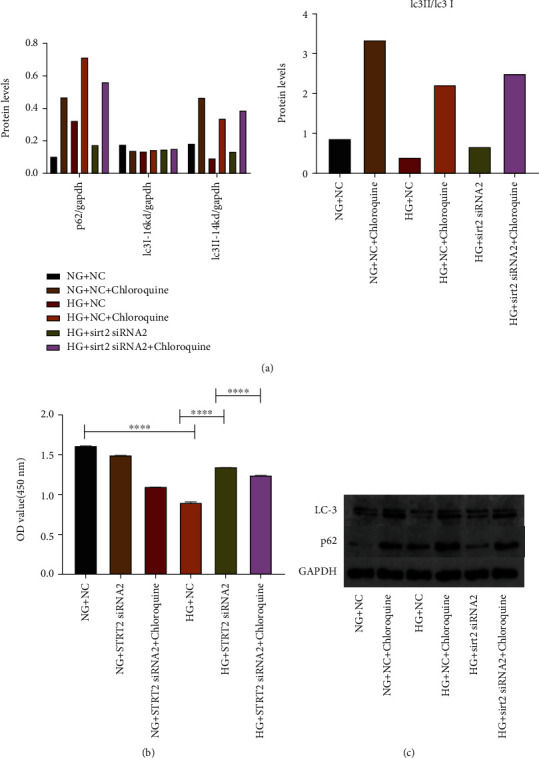
SIRT2 affects cellular proliferation by regulating cellular autophagy. (a) Histogram of expression of autophagy-related proteins. (b) Cell proliferation in each group. (c) Expression of autophagy-related proteins. Note: ^∗∗∗∗^*p* < 0.0001 for comparison between groups.

We observed that chloroquine inhibited the autophagy of cells in our experiments, so we treated the cells with chloroquine to observe its effect on cell proliferation. The results showed that cell proliferation was significantly reduced with high glucose, and there was an increase in cell proliferation after knocking down SIRT2, but cell proliferation was reduced after chloroquine challenge (*p* < 0.05) (Figure 5).

### 5.6. SIRT2 Affects Apoptosis by Regulating Cellular Autophagy

**Figure 6:**
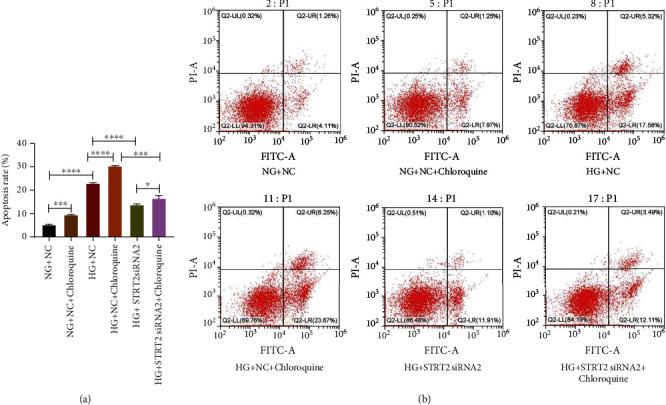
SIRT2 affects apoptosis by regulating cellular autophagy. (a) Apoptosis rate of each group. (b) Apoptosis flow cytogram. Note: ^∗^*p* < 0.05 for the contrast between groups; ^∗∗∗^*p* < 0.001 for the contrast between groups; ^∗∗∗∗^*p* < 0.0001 for the contrast between groups.

We also assessed the effect of chloroquine treatment and the knockdown of SIRT2 on apoptosis. The results showed that the apoptosis rate was elevated remarkably with excessive GLU in contrast to normal GLU, and apoptosis was more pronounced after chloroquine treatment. However, abrogation of STRT2 expression induced a remarkable reduction in apoptosis (*p* < 0.05). This suggests that SIRT2 may affect apoptosis by regulating cellular autophagy and thus apoptosis (Figure 6).

## 6. Discussion

The SIRT family, which includes SIRT1–SIRT7, is composed of 7 diverse types of HDACs. They have different subcellular localisations but are primarily located in the nuclei, mitochondrion, and cytoplasm. Almost all SIRTs are involved in cellular metabolic activities and modulate various cell functions [12, 13].

SIRT5 is an emerging cancer marker by which oncocytes can realize the reconfiguration of the metabolism for the purpose of supporting the anabolism of fast cellular fission [14]. SIRT5 expression is higher in hepatocellular carcinoma cell lines than in normal hepatocyte cell lines, and SIRT5 overexpression promotes hepatocellular carcinoma cell proliferation, but knockdown of SIRT5 inhibits hepatocellular carcinoma cell proliferation. Further, the knockdown of SIRT5 can promote apoptosis in hepatocellular carcinoma cells through the mitochondrial pathway [15]. SIRT5 is also responsible for growth and drug tolerance in mankind NSCLC. Its mRNA levels positively correlate with Nrf2 expression in lung cancer tissues, and knockdown of its expression reduces the expression of Nrf2 and its downstream drug resistance genes [16]. SIRT2, a NAD+-dependent class III histone deacetylase, is associated with cancer, genomic instability, and the pathogenesis of bacterial infection [17, 18]. The viral protein HBx can upregulate SIRT2 by targeting its promoter, indicating that SIRT2 promotes hepatitis B virus transcription and replication, thus promoting hepatitis B virus-mediated hepatocellular carcinoma [19]. SIRT2 is overexpressed in melanoma, and inhibition of SIRT2 reduces melanoma cell growth and clone formation [20, 21].

In the present study, we found that SIRT5 was not differentially expressed in high and normal glucose, while SIRT2 was upregulated in high glucose. We further observed that cell proliferation was decreased, and apoptosis was significantly increased in high glucose, but knockdown of SIRT2 resulted in a significant enhancement of cell proliferative activities and a decrease in apoptosis. Past researches revealed that SIRT2 could treat type 2 diabetes by regulating mitochondrial quality control [22]. Combined with the findings of the present study, this suggests that SIRT2 may act as a pathogenic factor in diabetic nephropathy and contribute to the progression of the disease.

Autophagy refers to a set of pathways through which cytoplasmic material is delivered to lysosomes for degradation [23]. Physiologically, autophagy is pivotal for modulating organismal developmental process, collaborating with the acquired immunosystem, sustaining energy homeostasis, and sustaining protein and cell organ quality regulation via eliminating protein and cell organ damages in the process of stress and senescence [24]. Autophagy also occupies a central position in the biology of most eukaryotes, often interacting with core metabolism, damage control, and the regulation of cell death [25]. Sirtinol, a strong SIRT1 suppressor, was discovered to trigger autophagy-related cellular death within MCF-7 cells [26]. P2Y2R is a purinergic receptor whose activation is involved in the etiopathogenesis of renal illness. P2Y2R can lead to AKT and SIRT1/FOXO3a-mediation autophagic function disorder, which in turn promotes the development of diabetic nephropathy [27]. Recent studies have shown that SIRT2 sustains mitochondrion biology and promotes cellular survival via modulating autophagic activities and mitochondrial autophagy [28, 29]. In the present study, our results showed that SIRT2 can affect the level of autophagy in cells. Furthermore, by subjecting the cells to chloroquine treatment, we found that cell proliferation was significantly reduced at high glucose levels, and there was an increase in cell proliferation after knocking down SIRT2, but cell proliferation was reduced after chloroquine treatment. SIRT2 can affect cellular autophagy, probably due to the fact that SIRT2 serves as a microtubule protein deacetylase regulating the acetylization of the microtubule network. Microtubule disassembly impairs autophagy, whereas inhibition of SIRT2 leads to microtubule network reconstitution and facilitates axon transportation and fusion of autophagy vesicles with lysosomes [30]. Nevertheless, the exact causal link still needs to be elucidated.

To sum up, SIRT2 expression was upregulated in MPC-5 cells after high-glucose treatment, and by knocking down the expression level of SIRT2, we found that it modulated cell biological functions. Overall, our findings suggest that SIRT2 may affect cell proliferation and apoptosis by regulating cell autophagy.

## Figures and Tables

**Table 1:** Primer sequence list.

	Forward (5′-3′)	Reverse (5′-3′)
GAPDH	ATGGGTGTGAACCACGAGA	CAGGGATGATGTTCTGGGCA
Sirt2	GGTGGAGAGGCAGAGATGGAC	TAGTGGCAGATGGTTGGCTTG

**Table 2:** Primer sequences used for construction of SIRT2 shRNA and shControl for SIRT2 RT-PCR.

Plasmid	Sequence (5′-3^″^)
shSIRT2-#2	GCCAACCATCTGTCACTACTT
shSIRT2-#3	CCTGCTCATCAACAAGGAGAA
shControl	GCAACAAGATGAAGAGCACCAA
SIRT2 forward	ATGGACTTCCTGTGGAGACTTA
SIRT2 reverse	GCCCGGCTATTCGCCCAGGG

## Data Availability

The data used to support the findings of this study are included within the article.

## Abbreviations

WB: Western blot
INS: Insulin
GLU: Glucose
T2D: Type 2 diabetes
T1D: Type 1 diabetes
FBS: Foetal bovine serum
PNC: Penicillin
RT: Room temperature
PFA: Paraformaldehyde
HDACs: Histone deacetylases
NSCLC: Non-small-cell lung cancer
RNase: Ribonucleas.

## References

[1] Zhang X. X., Kong J., Yun K. (2020). Prevalence of diabetic nephropathy among patients with type 2 diabetes mellitus in China: a meta-analysis of observational studies. *Journal of Diabetes Research*.

[2] Tsalamandris S., Antonopoulos A. S., Oikonomou E. (2019). The role of inflammation in diabetes: current concepts and future perspectives. *European Cardiology Review*.

[3] Magee C., Grieve D. J., Watson C. J., Brazil D. P. (2017). Diabetic nephropathy: a tangled web to unweave. *Cardiovascular Drugs and Therapy*.

[4] Flyvbjerg A. J. N. R. N. (2017). The role of the complement system in diabetic nephropathy. *Nature Reviews. Nephrology*.

[5] Wang G., Ouyang J., Li S. (2019). The analysis of risk factors for diabetic nephropathy progression and the construction of a prognostic database for chronic kidney diseases. *Journal of Translational Medicine*.

[6] Tung C. W., Hsu Y. C., Shih Y. H., Chang P. J., Lin C. L. (2018). Glomerular mesangial cell and podocyte injuries in diabetic nephropathy. *Nephrology*.

[7] Huang H., Zhang D., Wang Y. (2018). Lysine benzoylation is a histone mark regulated by SIRT2. *Nature Communications*.

[8] Hu F., Sun X., Li G. (2018). Inhibition of SIRT2 limits tumour angiogenesis via inactivation of the STAT3/VEGFA signalling pathway. *Cell Death & Disease*.

[9] Zhang L., Kim S., Ren X. (2020). The clinical significance of SIRT2 in malignancies: a tumor suppressor or an oncogene?. *Frontiers in Oncology*.

[10] Kozako T., Mellini P., Ohsugi T. (2018). Novel small molecule SIRT2 inhibitors induce cell death in leukemic cell lines. *BMC Cancer*.

[11] Huang S., Zhao Z., Tang D. (2017). Downregulation of SIRT2 inhibits invasion of hepatocellular carcinoma by inhibiting energy metabolism. *Translational Oncology*.

[12] Liu M., Yu J., Jin H. (2021). Bioinformatics analysis of the SIRT family members and assessment of their potential clinical value. *Oncotargets and Therapy*.

[13] Vargas-Ortiz K., Pérez-Vázquez V., Macías-Cervantes M. H. (2019). Exercise and sirtuins: a way to mitochondrial health in skeletal muscle. *International Journal of Molecular Sciences*.

[14] Bringman-Rodenbarger L. R., Guo A. H., Lyssiotis C. A., Lombard D. B. (2018). Emerging roles for SIRT5 in metabolism and cancer. *Antioxidants & Redox Signaling*.

[15] Zhang R., Wang C., Tian Y. (2019). SIRT5 promotes hepatocellular carcinoma progression by regulating mitochondrial apoptosis. *Journal of Cancer*.

[16] Lu W., Zuo Y., Feng Y., Zhang M. J. T. B. (2014). SIRT5 facilitates cancer cell growth and drug resistance in non-small cell lung cancer. *Tumour Biology*.

[17] Romeo-Guitart D., Leiva-Rodríguez T., Espinosa-Alcantud M. (2018). SIRT1 activation with neuroheal is neuroprotective but SIRT2 inhibition with AK7 is detrimental for disconnected motoneurons. *Cell Death & Disease*.

[18] González-Fernández R., Martín-Ramírez R., Rotoli D. (2019). Granulosa-lutein cell Sirtuin gene expression profiles differ between Normal donors and infertile women. *International Journal of Molecular Sciences*.

[19] Yu H. B., Jiang H., Cheng S. T., Hu Z. W., Ren J. H., Chen J. (2018). AGK2, a SIRT2 inhibitor, inhibits hepatitis B virus replication in vitro and in vivo. *International Journal of Medical Sciences*.

[20] Wilking-Busch M. J., Ndiaye M. A., Liu X., Ahmad N. (2018). RNA interference-mediated knockdown of SIRT1 and/or SIRT2 in melanoma: identification of downstream targets by large-scale proteomics analysis. *Journal of Proteomics*.

[21] Lim J., Son J., Ryu J., Kim J.-E. (2020). SIRT2 affects primary cilia formation by regulating mTOR signaling in retinal pigmented epithelial cells. *International Journal of Molecular Sciences*.

[22] Xu J., Kitada M., Koya D. (2020). The impact of mitochondrial quality control by Sirtuins on the treatment of type 2 diabetes and diabetic kidney disease. *Biochimica et Biophysica Acta (BBA)-Molecular Basis of Disease*.

[23] Yim W. W. Y., Mizushima N. (2020). Lysosome biology in autophagy. *Cell Discovery*.

[24] Li X., He S., Ma B. (2020). Autophagy and autophagy-related proteins in cancer. *Molecular Cancer*.

[25] Galluzzi L., Green D. R. (2019). Autophagy-independent functions of the autophagy machinery. *Cell*.

[26] Wang J., Kim T. H., Ahn M. Y. (2012). Sirtinol, a class III HDAC inhibitor, induces apoptotic and autophagic cell death in MCF-7 human breast cancer cells. *International Journal of Oncology*.

[27] Dusabimana T., Kim S. R., Park E. J. (2020). P2Y2R contributes to the development of diabetic nephropathy by inhibiting autophagy response. *Molecular Metabolism*.

[28] Yang W., Gao F., Zhang P. (2017). Functional genetic variants within the SIRT2 gene promoter in acute myocardial infarction. *PLoS One*.

[29] Cacabelos R., Carril J. C., Cacabelos N. (2019). Sirtuins in Alzheimer's disease: SIRT2-related genophenotypes and implications for pharmacoepigenetics. *International Journal of Molecular Sciences*.

[30] Manjula R., Anuja K., Alcain F. J. (2021). SIRT1 and SIRT2 activity control in neurodegenerative diseases. *Frontiers in Pharmacology*.

